# Antimicrobial Study of Newly Synthesized Lanthanide(III) Complexes of 2-[2-hydroxy-3-methoxyphenyl]-3-[2-hydroxy-3-methoxybenzylamino]-1,2-dihydroquinazolin-4(3H)-one

**DOI:** 10.1155/2007/37348

**Published:** 2008-02-13

**Authors:** Kalagouda B. Gudasi, Vidyadhar C. Havanur, Siddappa A. Patil, Basavaraj R. Patil

**Affiliations:** Department of Chemistry, Karnatak University, Dharwad 580 003, India

## Abstract

New lanthanide(III) complexes with 2-[2-hydroxy-3-methoxyphenyl]-3-[hydroxyl-3-methoxybenzylamino]-1,2-dihydroquin- azoline-4(3H)-one (Hmpbaq) have been synthesized and characterized by elemental analysis, conductance measurements, magnetic susceptibilities, spectroscopic (IR, NMR, UV, EPR), and thermal studies. Molar conductance studies indicate 1 : 1 electrolytic behavior for these complexes. IR spectra indicate that Hmpbaq acts as a tridentate ligand coordinating through carbonyl oxygen, benzyl amine nitrogen, and deprotonated phenolic oxygen. TG and DTA studies of La(III) and Pr(III) complexes indicate the presence of two coordinated water molecules. Based on these studies, the complexes have been formulated as [La(mpbaq)_2_(H_2_O)_2_]·NO_3_, where Ln = La(III), Pr(III), Nd(III), Sm(III), Eu(III), Gd(III), Th(III), Dy(III), and Y(III). The ligand, lanthanide(III) salts, and the corresponding complexes have been simultaneously screened for their antibacterial and antifungal activities and compared with the drugs in use.

## 1. INTRODUCTION

Lanthanide ion is a subject of increasing interest in bioinorganic and coordination chemistry. A sustained research activity has been devoted to lanthanide complexes, because of their successful application as diagnostic tools in biomedical analysis as MRI contrast agents [1, 2]. They have been used even as effective catalysts for the hydrolytic cleavage of phosphate ester bonds [3]. Lanthanide complexes have been found to exhibit anticancer, and fungicidal properties also [4]. Quinazolines as a class of heterocyclic compounds contain the pyrimidine nucleus in their structures. A majority of them have great therapeutic significance due to their high biological activity. 4(4H)-quinazolines have been tested successfully against cancer and HIV virus [5, 6]. The synthetic analogues of quinazolines have been found to exhibit antimalarial [7], anti-inflammatory [8], and anticancer activity [9]. They show potent and specific inhibitory action against leukemia cells [10]. Sulfonate ester-containing quinazoline derivatives [11] were found to act as antimicrobial agents. Apart from the above, 2,3-disubstituted quinazoline-4-(3H)-ones have been reported for their wide variety of pharmacological activity [12, 13]. The title ligand Hmpbaq possesses multiple coordinating sites such as phenolic oxygen, carbonyl oxygen, nitrogen of the benzoyl amino group, and nitrogen of the quinazoline ring. It may act as a monodentate, bidentate, or tridentate species. We have observed its tridentating behavior with transition metal ions [14]. In continuation of our work on quinazolines, here we report the conformational changes in the ligand on coordination with lanthanides and the relative antimicrobial activity.

## 2. MATERIALS AND METHODS

### 2.1. Chemistry

All the solvents used were of analytical grade. Hydrazine hydrate, methyl 2-aminobenzoate, and o-vanillin were procured from Rankem, Merck, and Himedia, respectively. O-aminobenzoyl hydrazide was synthesized as reported earlier [14]. The lanthanide nitrates were obtained by heating lanthanide oxides (99.9%) (Indian Rare Earths Limited, Mumbai, India) with dilute nitric acid (50%) and evaporating the excessive acid.

**Syntheses**

**Scheme 1:**
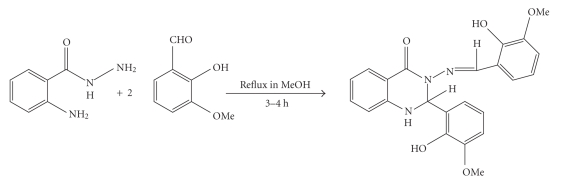
Synthetic route to the ligand Hmpbaq.

**Figure 1:**
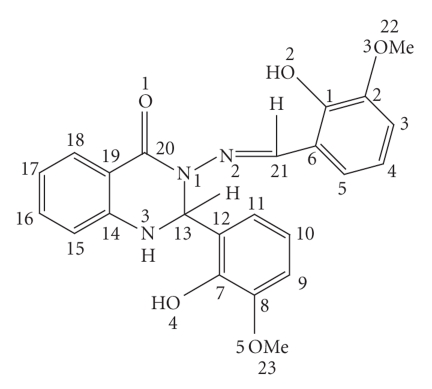
The numbering system of the ligand Hmpbaq.

The ligand Hmpbaq was synthesized as in our earlier report [14] (see Scheme 1). The numbering system of the ligand Hmpbaq is given in Figure 1. A solution of Ln(NO_3_)_3_ (1 mmol) and Hmpbaq 0.838 gm (2 mmol) was refluxed in ethanol (25 mL) for 2-3 hours. The pH of the solution was then raised to 6.5 by the addition of sodium acetate and refluxed further for an hour. The precipitate obtained after concentrating the solution was filtered off, washed with water, and dried in air (yield: 90%, mp
>245 °C).

**Physical measurements**

Elemental analysis was performed on a Carlo Erba Strumentazione (Milan, Italy) CHN analyzer. IR spectra were obtained on a Nicolet 170 SX FT-IR spectrometer using KBr pellets, in the range 400–4000 cm^−1^. ^1^H- and ^13^C NMR spectra were monitored on a JEOL-AMX-400 NMR spectrometer, using DMSO-d_6_ as the solvent. TG/DTA were recorded on a PerkinElmer (Mass, USA) (Pyris Diamond) analyzer in N_2_ atmosphere at a heating rate of 10°C. Mass spectra of the ligand and complex were recorded on a Thermofinnigan 1020-automated GCMS and JEOL SX 102/DA-6000 mass spectrometer/data system using argon and xenon (6 kv, 10 ma) as the FAB gas, respectively. UV-Visible spectra were obtained on a Hitachi 2001 spectrometer. EPR spectra of the Gd(III) and Tb(III) complexes were monitored on a Varian E-4X band spectrometer. Molar conductivities were obtained on an Elico conductivity bridge having platinum electrodes. Magnetic moments were measured with a Faraday balance using Hg[Co(NCS)_4_] as calibrant. Diamagnetic corrections were made using Pascal constants. The metal contents were determined by complexometric titrations with EDTA using xylene orange as an indicator.

### 2.2. Pharmacology

**Antibacterial activity**

The antibacterial activity of the ligand, metal salts, and the corresponding complexes were assayed simultaneously against *Pseudomonas aeruginosa* (PA), *Bacillus cirroflagellosus* (BC), by cup-plate method [15]. Nutrient broth was prepared by dissolving peptone (0.5%), yeast extract (0.15%), beef extract (0.15%), sodium chloride (0.36%), and monopotassium phosphate (0.13%) in distilled water (100 mL). The pH of the solution was adjusted to 7.2 by adding sodium hydroxide solution (4%) and the resulting solution was autoclaved for 20 minutes at 15 psi. One day prior to the experiment, the cultures of *Pseudomonas aeruginosa* and *Bacillus cirroflagellosus* were inoculated in nutrient broth (inoculation medium) and incubated overnight at 37°C. Nutrient agar medium was prepared by dissolving peptone (1%), yeast extract (0.6%), beef extract (0.5%), and sodium chloride (0.5%) in distilled water. The pH of the solution was adjusted to 7.2 by adding 4% aqueous sodium hydroxide solution. Agar (2.4%) was then added and the whole solution was autoclaved for 20 minutes at 15 psi. Each test sample (1 mg) was dissolved in DMSO (1 mL), and 0.1 mL of this solution (10 *μ*g) was used for testing. Inoculation medium containing 24-hours grown culture was added aseptically to the nutrient medium and mixed thoroughly to get the uniform distribution. This solution was poured (25 mL in each dish) into Petri dishes and then allowed to attain room temperature. Thereafter, punching the set of agar with a sterile cork borer and scooping out the punched part made the cups. The diameter of each cup was 5 mm. Norfloxacin was used as the standard and DMSO as the solvent control. The test samples and the standard were tested at a concentration of 10 *μ*g. The plates were allowed to stand for an hour in order to facilitate the diffusion of the drug solution. Then the plates were incubated at 37°C for 48 hours. The zones of inhibition against all the microorganisms were measured in millimeters.

**Antifungal activity**

The antifungal activity of the ligand and the corresponding metal complexes were tested against the pathogenic fungi *Aspergillus niger* (AN) and *Penicillium notatum* (PN) by cup-plate method. Nutrient agar medium was prepared by the same method as explained under evaluation of antibacterial activity. One and half day prior to the experiment, the fungal cultures of *Aspergillus niger* and *Penicillium notatum* prepared in the inoculation medium were incubated at 37°C for 36 hours. The fungal medium was prepared by dissolving peptone (0.5%), sodium chloride (0.36%), monopotassium phosphate (0.13%), and glucose (2%) in distilled water (100 mL). The pH of the solution was adjusted to 7.2 by adding sodium hydroxide solution (4%) and the resulting solution was autoclaved for 20 minutes at 15 psi. This was cooled to 45–50°C with gentle shaking. One and half day, grown cultures were added aseptically to this medium and mixed thoroughly to get uniform distribution. The solutions of the test samples and standard were evaluated for antifungal activity by cup-plate method at a concentration of 10 *μ*g. The zone of inhibition was measured in millimeter for the particular test sample with each organism at 48 hours interval. Griseofulvin was used as the standard.

## 3. RESULTS AND DISCUSSION

### 3.1. Chemistry

The analytical data (see Table 1) indicate that the complexes have 1 : 2 (metal : ligand) stoichiometry and can be represented by the general formula [La(mpbaq)_2_(H_2_O)_2_]⋅NO_3_. The lanthanide complexes are stable, nonhygroscopic, and yellow in color. They are soluble in DMSO and DMF, sparingly soluble in ethanol and methanol, but insoluble in benzene, ether, and chloroform. The molar conductance data (see Table 1) of the complexes in DMSO at 10^−3^ M is in the range of 44.00–49.00 ohm^−1^cm^2^mol^−1^, which indicates 1 : 1 electrolytic nature of the complexes.

### 3.2. Infrared spectra

The diagnostic IR frequencies of the ligand Hmpbaq and its complexes are compiled in Table 2. In the spectrum of the Hmpbaq, the strong band observed at 1653 cm^−1^ is assigned to *ν*(C=O) of the quinazoline ring. The appearance of this band at a lower wave number is due to the existence of strong intermolecular hydrogen bonding between the oxygen of C=O and the hydrogen of the 2-[2-hydroxy-3-methoxy phenyl) group, as observed in its crystal structure [14]. The band at 1610 cm^−1^ is assigned to benzyl amine *ν*(C=N). The two nonequivalent phenolic −OH groups are observed at 3460–2940 cm^−1^ as a strong unresolved broad band. A medium intensity band at 3280 cm^−1^ is assigned to *ν*(NH) of the quinazoline ring. In the spectra of all the complexes, the *ν*(C=O) has shifted to lower wave numbers indicating the involvement of carbonyl oxygen in coordination. The band due to *ν*(C=N) has shifted to lower energy (1616–1572 cm^−1^), indicating coordination through benzyl amine nitrogen. The band due to phenolic −OH groups was not observed in the spectra of the complexes, it might have been obscured by a broad band due to water. However, the coordination through only one or both of the phenolic oxygen/s was further confirmed by ^1^H NMR spectral studies. Strong band at 1381 cm^−1^ in the spectra of all the complexes indicates the presence of ionic nitrate. The presence of coordinated water molecules is suggested by the appearance of characteristic rocking frequency at 825 cm^−1^. This was further confirmed by thermal studies.

### 3.3. ^1^H NMR spectral studies

^1^H NMR spectra of the Hmpbaq and its La(III) complex were recorded in DMSO-d_6_ and the data along with the assignments are displayed in Table 3. Figure 1 gives the numbering system employed for ^1^H NMR assignments. The ^1^H NMR spectrum of Hmpbaq exhibits two sharp peaks at 11.31 and 9.42 ppm, corresponding to O(2)H (D_2_O exchangeable) and O(4)H (D_2_O exchangeable), respectively. Two singlets and one doublet at 8.43, 7.43, and 7.81 ppm are due to C(21)H, N(3)H (D_2_O exchangeable) and C(13)H, respectively. N(3)H and C(13)H, being cis to one another, coupled together and this coupling results in the splitting of C(13)H signal. Four triplets [C(17)H, C(16)H, C(10)H, and C(4)H] and six doublets [C(18)H, C(15)H, C(11)H, C(9)H, C(5)H, and C(3)H] have merged to give a multiplet at 7.31–6.6 ppm corresponding to ten aromatic protons. The resonance due to O(3)C(22)H_3_ and O(5)C(23)H_3_ appeared at 3.85 ppm. In the spectrum of La(III) complex, the resonance due to O(4)H has appeared at the same position as in the ligand (9.40 ppm) indicating the noninvolvement of O(4)H in the coordination. The singlet at 11.31 ppm in the ligand spectrum ascribed to O(2)H group is not observed in La(III) complex. This confirms the involvement of O(2)H in coordination with the metal ion via deprotonation. The other signals [C(21)H, N(3)H, C(13)H, C(17)H, C(16)H, C(10)H, C(4)H, C(18)H, C(15)H, C(11)H, C(9)H, C(5)H, C(3)H, O(3)C(22)H_3_, and O(5)C(23)H_3_] in La(III) complex did not show any considerable shift.

### 3.4. Magnetic and EPR spectral studies

The effective magnetic moments (see Table 1) of all the complexes indicate that they are paramagnetic in nature except La(III) and Y(III) which are diamagnetic. The values obtained are similar to the Van Vleck and Frank [16] and Hund's values except in case of Sm(III) and Eu(III) where slightly higher values were obtained. This is due to low *J-J* separation, which leads to thermal population of higher energy levels. The values obtained are similar to those of typical lanthanide ions [17] and indicate the noninvolvement of 4f electrons in bonding due to their very effective shielding by the 5s^2^ 5p^6^ octet. Gd(III) ion has 4f^7^ electronic configuration with ^8^S_7/2_ single-ion ground state. The energy level of the lowest excited state is very high with no contribution from orbital angular momentum and the anisotropic effect [18]. A “g” value of 1.99 (at room temperature) and 2.04 (at liquid nitrogen temperature) compared to the free-ion value of tetracynoethylene (2.00277) with broad resonance lines was obtained. The “g” values being almost the same and similar line widths indicate that the line widths are independent of temperature. Further, the complete absence of zero-field hyperfine splitting and the presence of broad bands indicate that the Gd(III) ion is located in a rather disordered environment caused by strain. These strains (caused by “g” strain for the “g” tensor distribution, D-strain for the zero field splitting distribution) arise due to random hydrogen bonds between water molecules and the complex leading to distortions, which lead to broad resonance EPR lines [19, 20].

### 3.5. UV-visible spectra

The electronic spectral data of the two representative complexes are given in Table 4. The free ligand shows an intense band at 32786 cm^−1^and two weak bands at 28328 and 38610 cm^−1^ of which the first two are assigned to the $\text{n}\to \pi^{*}$ and the latter to the $\pi \to \pi^{*}$ transitions, respectively. The electronic spectra of the complexes are dominated by ligand bands, with a slight shift to higher or lower energy levels. This slight shift was attributed to the effects of crystal field upon the interelectronic repulsion between the 4f electrons [21]. The bonding parameter (b^1/2^), Sinha's covalence parameter (*δ*), nephelauxetic parameter (*β*), and angular covalence (*η*) have been calculated according to literature procedure [22, 23]. *β* values being less than unity and positive value of b^1/2^ and *δ* indicate metal-ligand covalent bonding. According to Karraker [24], the shape of the hypersensitive transition reflects the environment of the metal ion. On comparison of the spectra with that of known compounds, it is concluded that the coordination number of the present complexes is eight.

### 3.6. Thermal studies

The TG/DTA study of La(III) and Pr(III) complexes was carried out as the representative. In case of La(III) complex, the initial weight loss of 11.24% (Cal. 11.34%) at 250°C corresponds to the loss of two water molecules. The temperature ranges suggest the presence of coordinated water molecules. The next weight loss in the temperature range of 250–350°C is due to the loss of one ionic nitrate molecule (Obs. 10.00%; Cal. 10.35%). A further weight loss in the range of 350–800°C corresponds to two ligand molecules (Obs. 69.00%; Cal. 69.89%). Finally, the most stable oxide was formed, on further heating up to 1000°C. The percentage of metal obtained is in confirmation with the analytical values for the metal content.

In case of Pr(III), the thermogram exhibits a loss of 3.00% (Cal. 3.00%) corresponding to the loss of two coordinated water molecules. A loss of 10.00% (Cal. 10.35%) between 240–260°C is due to the removal of one ionic nitrate molecule. A further weight loss of 69.00% (Cal. 69.72%) is due to two ligand molecules. Finally, the most stable oxide Pr_6_O_11_ is formed.

### 3.7. Mass spectral studies

In the present investigation, the mass spectrum of Hmpbaq shows the formation of molecular ion peak at m/z = 419 and corresponds to the total molecular weight of the ligand. The FAB mass spectra of [Sm(mpbaq)_2_(H_2_O)_2_]⋅NO_3_ and [Eu(mpbaq)_2_(H_2_O)_2_]⋅NO_3_ show the molecular ion peaks at m/z = 1084 and 1086, respectively, supporting the composition of the complexes.

### 3.8. Pharmacology

The results of the antibacterial and antifungal studies are given in Table 5. The ligand Hmpbaq was less active against PN, PA, and BC except against AN where it has shown moderate activity. Compared to the ligand, the complexes were moderately active against PA. All the complexes, except La(III), Pr(III), and Nd(III) complexes, show moderate activity against BC. It was observed that, compared to the ligand and metal salts, the complexes exhibited enhanced antibacterial activity, which is due to the synergistic effect that increases the lipophilicity of the complexes. Chelation decreases the polarity of the metal ion, which further leads to the enhancement of complex's lipophilicity. Since the microorganism cell is surrounded by a lipid membrane which favors the passage of lipid soluble materials, increased lipophilicities allows the penetration of complex into and through the membrane and deactivates the active enzyme sites of the microorganisms [25].

## 4. CONCLUSION

Based on the above data, structure I is proposed for the lanthanide(III) complexes having the general formula [Ln(mpbaq)_2_(H_2_O)_2_]⋅NO_3_, where Ln = La(III), Pr(III), Nd(III), Sm(III), Eu(III), Gd(III), Tb(III), Dy(III), and Y(III). In the present complexes, due to rotation about the N–N bond, the ligand changes its conformation to facilitate the coordination in ONN fashion as in the case of Cd(II) complex as reported earlier [14]. The antibacterial activity of the ligand is enhanced on complexation, whereas no distinct change is observed in case of antifungal activity.

## Figures and Tables

**Figure 2:**
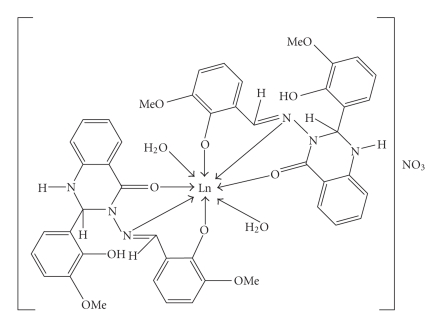
Proposed structure of lanthanide(III) complex.

**Table 1:** Elemental analysis magnetic and conductance data of Hmpbaq and its Ln(III) complexes.

Sl. No.	Compounds	Found (calculated) %				Magnetic moments	Molar conductance Ohm^−1^cm^2^mol^ −1^
		M	C	H	N		
1	Hmpbaq (C_23_H_21_N_3_O_6_)	—	65.86	5.03	10.03	—	—
			(65.87)	(5.01)	(10.02)		
2	[La(mpbaq)_2_(H_2_O)_2_]⋅NO_3_	12.90	51.45	4.00	9.10	Dia	44.58
		(12.94)	(51.44)	(4.10)	(9.13)		
3	[Pr(mpbaq)_2_(H_2_O)_2_]⋅NO_3_	13.00	51.30	4.15	9.15	3.86	45.28
		(13.10)	(51.35)	(4.09)	(9.11)		
4	[Nd(mpbaq)_2_(H_2_O)_2_]⋅NO_3_	13.35	51.25	4.01	9.16	3.52	47.23
		(13.37)	(51.19)	(4.08)	(9.08)		
5	[Sm(mpbaq)_2_(H_2_O)_2_]⋅NO_3_	13.85	50.85	4.10	9.06	1.70	47.58
		(13.86)	(50.90)	(4.05)	(9.03)		
6	[Eu(mpbaq)_2_(H_2_O)_2_]⋅NO_3_	13.95	50.75	4.00	8.98	3.80	48.02
		(13.99)	(50.83)	(4.05)	(9.02)		
7	[Gd(mpbaq)_2_(H_2_O)_2_]⋅NO_3_	14.45	50.55	4.00	9.00	7.90	47.28
		(14.41)	(50.58)	(4.03)	(8.98)		
8	[Tb(mpbaq)_2_(H_2_O)_2_]⋅NO_3_	13.60	50.60	4.10	8.95	9.80	49.58
		(14.54)	(50.50)	(4.02)	(8.96)		
9	[Dy(mpbaq)_2_(H_2_O)_2_]⋅NO_3_	14.90	50.34	4.08	8.90	10.82	49.20
		(14.81)	(50.34)	(4.01)	(8.93)		
10	[Y(mpbaq)_2_(H_2_O)_2_]⋅NO_3_	8.70	53.98	4.25	9.55	Dia	47.50
		(8.69)	(53.96)	(4.30)	(9.58)		

**Table 2:** Diagnostic IR frequencies of the ligand Hmpbaq and its complexes.

Sl. No.	Compound	*ν*(OH) of water	*ν*(NH)	Quinazoline *ν*(C=O)	Phenolic *ν*(OH)	*ν*(C=N)	IonicNO_3_
1	Hnpbaq	3518b	3314 s	1653s	3065b	1616s	—
2	[La(mpbaq)_2_(H_2_O)_2_]⋅NO_3_	3425b	no^a^	1640s	no^b^	1572sh	1381m
3	[Pr(mpbaq)_2_(H_2_O)_2_]⋅NO_3_	3428b	no^a^	1638s	no^b^	1585sh	1385m
4	[Nd(mpbaq)_2_(H_2_O)_2_]⋅NO_3_	3433b	no^a^	1633s	no^b^	1585sh	1383m
5	[Sm(mpbaq)_2_(H_2_O)_2_]⋅NO_3_	3430b	no^a^	1633s	no^b^	1585sh	1384m
6	[Eu(mpbaq)_2_(H_2_O)_2_]⋅NO_3_	3451b	no^a^	1634s	no^b^	1586sh	1385m
7	[Gd(mpbaq)_2_(H_2_O)_2_]⋅NO_3_	3429b	no^a^	1633s	no^b^	1586sh	1384m
8	[Tb(mpbaq)_2_(H_2_O)_2_]⋅NO_3_	3439b	no^a^	1634s	no^b^	1586sh	1385m
9	[Dy(mpbaq)_2_(H_2_O)_2_]⋅NO_3_	3438b	no^a^	1633s	no^b^	1586sh	1387m
10	[Y(mpbaq)_2_(H_2_O)_2_]⋅NO_3_	3428b	no^a^	1633s	no^b^	1586sh	1349m

s-strong, m-medium, b-broad, w-weak.

no^a^—not observed distinctly, might have been observed by the broad band of water molecules.

no^b^—not observed distinctly, might have been observed by the broad band of water molecules.

**Table 3:** ^1^H NMR spectral data of Hmpbaq and its La(III) complex.

Chemical shift in ppm		
Protons	Hmpbaq	La(III) complex
O(3)H	11.31 (s,1H)	—
O(4)H	9.42 (s,1H)	9.42 (s,1H)
C(21)H	8.43 (s,1H)	8.46 (s,1H)
C(13)H	7.81 (d,1H, J = 7.52 Hz)	7.88 (d,1H, J = 7.52 Hz)
N(3)H	7.43 (s,1H)	7.58 (s,1H)
C(13)H–C(5)H, C(9)H–C(11)H	7.31–6.60 (m,10 Ar–H)	7.38–6.37 (m,10 Ar–H)
and C (15)H–C(18)H,		
O(3)C(22)H, and O(5)C(23)H_3_	3.80 (s,6H)	3.82 (s,6H)

s = singlet; d = doublet; m = multiplet; Ar–H = Aromatic protons.

**Table 4:** UV-visible spectral data of the ligand Hmpbaq, Pr(III), and Sm(III) complexes.

Complex	Assignment	$\nu_{\text{max}}$ of Ln^+3^ ion in cm^−1^	$\nu_{\text{max}}$ of complexes in cm^−1^	*β*	Related parameter
[Pr(mpbaq)_2_(H_2_O)_2_]⋅NO_3_	$\text{n}\to \pi^{*}$	25142	25062	0.99681	δ=0.46919%
	$\pi \to \pi^{*}$	31237	31152	0.99686	b^1/2^ = 0.048321
		38610	38314	$\frac{\text{0}\text{.99232}}{\beta \text{=0}\text{.99533}}$	η=0.06836
[Sm(mpbaq)_2_(H_2_O)_2_]⋅NO_3_	$\text{n}\to \pi^{*}$	25642	25445	0.99231	δ=0.72522%
	$\text{n}\to \pi^{*}$	31250	31250	0.99376	b^1/2^ = 0.06
		38610	38314	$\frac{\text{0}\text{.99233}}{\beta \text{=0}\text{.99280}}$	η=0.08791

**Table 5:** Inhibitory activity of Hmpbaq and its Ln(III) complexes.

Compound	Antifungal		Antibacterial	
	PN	AN	PA	BC

Hnpbaq	+	++	+	+
[La(mpbaq)_2_(H_2_O)_2_]⋅NO_3_	+	+	++	+
[Pr(mpbaq)_2_(H_2_O)_2_]⋅NO_3_	+	+	++	+
[Nd(mpbaq)_2_(H_2_O)_2_]⋅NO_3_	+	+	++	+
[Sm(mpbaq)_2_(H_2_O)_2_]⋅NO_3_	+	+	++	+ +
[Eu(mpbaq)_2_(H_2_O)_2_]⋅NO_3_	+	+	++	+ +
[Gd(mpbaq)_2_(H_2_O)_2_]⋅NO_3_	+	+	++	+ +
[Tb(mpbaq)_2_(H_2_O)_2_]⋅NO_3_	+	+	++	+ +
[Dy(mpbaq)_2_(H_2_O)_2_]⋅NO_3_	+	+	++	+ +
[Y(mpbaq)_2_(H_2_O)_2_]⋅NO_3_	+	+	++	+ +
Grisofulvin	+++	+++	−	−
Norfloxacin	−	−	+++	+ ++

Key to interpretation (−) = no inhibition zone = inactive; 1–5 mm(+) = less active; 6–10 mm(++) = moderately active; 10–15 mm(+++) = highly active

PN = Penicillium notatum; AN = Aspergillus niger; PA = Pseudomonas aeruginosa; BC = Bacillus cirroflagellosus.
